# Impact of Employment, Essential Work, and Risk Factors on Food Access during the COVID-19 Pandemic in New York State

**DOI:** 10.3390/ijerph18041451

**Published:** 2021-02-04

**Authors:** Lauren A. Clay, Stephanie Rogus

**Affiliations:** 1 Department of Health Administration and Public Health, D’Youville College, Buffalo, NY 14201, USA; 2 School of Global Public Health, New York University, New York, NY 10003, USA; 3 Department of Family and Consumer Sciences, New Mexico State University, Las Cruces, NM 88003, USA; srogus@nmsu.edu

**Keywords:** COVID-19, employment, essential workers, food access, food security

## Abstract

The COVID-19 pandemic disrupted food systems and the economy in the U.S. and abroad. This cross-sectional study examined the direct and indirect impacts of COVID-19 on food access among low-income and Black, Indigenous, and people of color (BIPOC) in New York State. New York residents were recruited to complete a web-based survey through Qualtrics. The survey took place in May and June 2020 and asked participants about COVID-19 health impacts, risk factors, and food access. Chi-square analysis examined issues with food access experienced by demographic characteristics, work disruptions, health impacts, and household risk for contracting the virus and experiencing severe illness, and significant results were analyzed in a series of logistic regression models. After accounting for covariates, Hispanic respondents, those with likely Major Depressive Disorder, and essential workers were more likely to experience worse food access during COVID-19. Improved policies and services to address impacts on vulnerable populations such as BIPOC, those suffering from mental health disorders, and workers in lower-paying essential jobs can reduce the risk of food access issues at this time. Future research can identify how food access issues during the pandemic influenced diet quality, chronic disease risk and infection, and persistence of food access issues.

## 1. Introduction

The coronavirus 2019 (COVID-19) pandemic impacted countries across the world, including the United States. As of early January 2021, over 86 million COVID-19 cases and close to 2 million deaths have been reported around the globe [1]. The U.S. alone has seen more than 21 million cases and over 350,000 deaths, which account for nearly 20% of the confirmed cases and deaths worldwide [2]. 

The pandemic disrupted the economy and exacerbated vulnerabilities in the food system. Unemployment rates early in the pandemic rose to levels not seen since the Great Depression and stood at 7.9% in September 2020 [3]. Employment loss and disruption led to reductions in food access for many Americans. Stockpiling and hoarding created food supply shortages and stay-at-home orders reduced grocery shopping trips and eating out, further limiting access to food. Across the country, almost 14% of adults reported not having enough to eat in the last seven days in early December 2020 compared to the pre-pandemic rate of 3.7% for the previous year (2019) [3]. Other studies have reported increases in food insecurity rates in various parts of the country during the pandemic [4,5,6]. 

Food access and food security are closely related concepts. Food security is defined as “access by all people at all times to enough food for an active, healthy life” [7]. This includes the ability to acquire a sufficient quantity and quality of culturally acceptable foods in socially acceptable ways that alleviates worry and feelings of deprivation [8]. Food access is one component of food security that is important to explore given the unique disruptions in the food system experienced during COVID-19.

Groups at higher risk of contracting the virus or experiencing severe illness include essential workers, older adults, and adults with chronic conditions such as cardiovascular disease, diabetes, chronic respiratory disease, hypertension, and cancer [9,10]. Essential workers perform vital functions for society and work in industries that include healthcare, transportation, defense, information technology, food and agriculture, and public works, among others [10,11,12,13,14,15,16]. Essential workers are disproportionately likely to be less educated and Black, Indigenous, and people of color (BIPOC) and to earn lower wages than workers in other industries [10,11,16]. Some essential workers experienced job loss due to reduced operations during the pandemic, which put these workers at greater risk of both contracting the virus and experiencing a loss of income due to the pandemic [3,10,11,17]. Therefore, it comes as no surprise that COVID-19 infection and deaths in the U.S. have been disproportionately high among BIPOC [18]. These populations also experienced the indirect effects of the pandemic, including job loss or disruption and food insecurity, at higher rates than other Americans [3].

Several studies have reported higher rates of loneliness and depression during the pandemic [19,20,21]. The impacts of COVID-19 on mental health are likely due to the psychological suffering associated with the risk of contracting the disease and the secondary effects of the pandemic, including economic instability, loneliness, and social isolation [19,22]. Depression and anxiety are particularly likely for those who have contracted COVID-19 or are at higher risk of exposure to the virus and for those with concerns about job and financial security due to COVID-19-related job disruptions.

The purpose of this study is to understand the impact of COVID-19 on food access. It examines the impact of contracting the virus or knowing someone who has contracted the virus, employment disruptions, essential worker status, being at high risk of experiencing severe illness, and likely depression and anxiety on food access in New York State. This study oversampled less educated, low-income, and BIPOC to target groups believed to be most vulnerable to COVID-19 and its indirect effects.

## 2. Materials and Methods

### 2.1. Sample

A purposive quota sample of 415 individuals in New York State, excluding New York City (NYC), were recruited to complete a web survey about their experiences with the pandemic including food access and health impacts. Quotas were set for 50% Black or African American, 50% Hispanic, and 50% low-education (high school or less) or low household income (less than $50,000) to oversample individuals experiencing a greater disease burden with COVID-19 and also at increased risk for food insecurity or chronic disease burden. The NYC metro area was excluded from this analysis as the context for NYC was very different from that for the rest of the state. New York City experienced an early surge of COVID-19 with a significant impact on the healthcare systems and citywide activities [23,24,25], whereas statewide closure of non-essential businesses during NY on Pause was driven by the experience in NYC and other parts of the state did not have the same surge, disease burden, and impact [24]. NY on Pause was the Executive Order signed by Governor Cuomo outlining a 10-point plan for ensuring the safety of New Yorkers during the COVID-19 pandemic, including limiting the operation of in-person work for non-essential business [26].

### 2.2. Data Collection

Cross-sectional data were collected through a web survey adapted from the validated survey developed by the National Food Access Research Team (NFACT) [27,28]. Questions about food access were adopted directly from the NFACT survey, which achieved an alpha value of 0.70 [27]. Questions about employment impact, health, and risk factors were adapted from the PhenX COVID-19 Toolkit [29,30,31,32]. Data collection took place between 15 May and 20 July 2020 during statewide phased reopening from NY on Pause [25,26,33]. The complete survey is published and available in the COVID-19 and Social Determinants of Health Data Collection Instrument Repository [34]. Potential participants were provided a consent statement and were asked to confirm they were aged 18 or older and consented to participate before commencing with the survey. The survey research firm Qualtrics recruited the quota-based sample from survey panels maintained by the firm. The median time for survey completion was 13 min. The survey asked about COVID-19 impacts, food access, determinants of health, perceptions of the response, and individual and household characteristics. Respondents that completed the survey faster than half the median time were automatically excluded for poor data quality [35,36]. Each completed survey was reviewed for poor data quality due to straight-lined responses and non-sense answers following Qualtrics recommended data cleaning practices [35,36]. The D’Youville College Institutional Review Board reviewed and approved this research as Exempt prior to commencing data collection. 

### 2.3. Measures

#### 2.3.1. Food Access

Food access was measured by asking respondents how often since the COVID-19 pandemic began they (1) could not afford the amount or kind of food their household wanted to buy, (2) could not find as much food as they wanted to buy, (3) could not find the quality of food their household preferred to eat, and could not get food through a (4) food pantry or soup kitchen, (5) school food program, and (6) community meal program [37]. Each item was scored with a 1 assigned for reporting ever (sometimes, most of the time, always) having the issues and a 0 assigned for never (never) having the issue. Items were summed for a food access score (range 0–6). Using the mean as a cut point (mean 2.96), each respondent was classified as having better (score 0–2) or worse (score 3–6) food access. 

#### 2.3.2. Employment Impact

Employment impact was assessed by asking respondents if they experienced an employment change since the COVID-19 pandemic began (lost job, reduced income or hours, furloughed, work from home, increased hours, or no job changes). Respondents indicating a job loss, furlough, or reduced income or hours were classified as having reduced work.

#### 2.3.3. Health Impact

COVID-19 burden and mental health were assessed to understand the health impact of the pandemic in the study sample. COVID-19 disease burden was assessed by asking respondents to indicate whether they themselves or a family member, friend, or someone else they knew had ever quarantined, tested positive, been hospitalized, or died from COVID-19. Any respondent indicating that they had this experience personally was classified as having a “self COVID-19 impact” and any respondents indicating that they had a family, friend, or someone else they knew that had one of those experiences was classified as having a “direct COVID-19 impact”. Mental health was assessed using two validated screeners for anxiety and depression. Likely Generalized Anxiety Disorder was assessed using the GAD-2, which asks about feeling nervous, anxious, on edge, or worried. Standardized scoring (sensitivity 86%, specificity 83%) of the two-items was computed, and the recommended cut point of 3 was used to classify participants as having likely Generalized Anxiety Disorder [38]. Likely Major Depressive Disorder was assessed using the PHQ-2, which asks about interest or pleasure in doing things and feeling down, depressed, or hopeless. Standardized scoring (sensitivity 83%, specificity 92%) of the two-items was computed, and the recommended cut point of 3 was used to classify participants as having likely Major Depressive Disorder [39].

#### 2.3.4. Risk Groups

Risk groups for COVID-19 included essential workers due to increased exposure, high-risk households or those households with a member with increased risk of severe illness due to older age or a high-risk medical condition, and individuals with a pre-existing chronic health condition. Essential worker status was determined by asking respondents to indicate (yes/no) if they were required to work outside of the home during statewide stay-at-home orders. High-risk households were classified by asking respondents if anyone in their household was at high risk for COVID-19 complications due to a health condition or older age (yes/no) [40]. Pre-existing health conditions were assessed by asking respondents to check all that apply on a list of chronic conditions (any/none).

#### 2.3.5. Sociodemographic Characteristics

Demographic information was collected about gender (female, transgender, non-binary, other, male), household annual income in 2019 (<$13,000, $13,000–24,999, $25,000–49,999, $50,000–74,999, $75,000+), age (18–24, 25–44, 45–64, 65+), education (high school or less, some college, associate’s degree, technical school, bachelor’s degree, graduate degree), and race and ethnicity (non-Hispanic White, Black or African American, Hispanic, other or more than one race).

### 2.4. Data Analysis

Using a model building approach, bivariate analysis of employment impact, health, risk, and sociodemographic characteristics were examined using a chi-square analysis with the outcome food access [41]. Each factor independently associated with the outcome food access was included in a multivariate analysis. A series of four logistic regression models were fit. The first examined job impact to understand the influence of a change in economic circumstances on food access. The second model added COVID-19 disease burden of the respondent and their social network and mental health. The third model added COVID-19 risk factors including race and ethnicity, essential worker, and high-risk household status. The fourth and final model added sociodemographic characteristics. Adjusted odds ratios and 95% confidence intervals are reported. All statistical analyses were conducted using Stata version 16 (StataCorp, College Station, TX, USA) [42].

## 3. Results

The sample was 56.6% female, non-binary (0.24%), or other (0.96%) (Table 1). One third of the sample reported an annual income in 2019 of $25,000 or less (35.6%), one quarter reported an annual income of $25,000–$50,000, and nearly 25% reported an annual income of over $75,000. The mean age of the sample was 38 years old. Nearly one third of respondents reported a completed high school education or less (31.6%), nearly 40% reported some college including Associate’s degree and technical school, and the remaining 30% reported a Bachelor’s or graduate degree. Three-quarters of respondents reported Black or African American race (31.6%) or Hispanic ethnicity (40.6%), and 20.6% of participants were non-Hispanic White.

When looking at the frequency of COVID-19 impacts on the sample, 40.7% reported reduced work due to the pandemic including furlough, job loss, or reduced hours or income. Most study participants indicated they had some experience (quarantined (17.8%), tested positive (3.9%), or hospitalized (3.2%)) with COVID-19 themselves or that a friend, family member, or another person they knew had quarantined, tested positive, was hospitalized, or died due to the virus (62.2%). Mental health impacts were also widespread, with 30.5% of respondents screening for likely Generalized Anxiety Disorder and 41.2% screening for likely Major Depressive Disorder.

Different risk groups were examined in the sample. More than one third of respondents reported being an essential worker (36.4%), and 41.2% of study participants were high-risk households with someone aged 65 or older or with a high-risk medical condition residing within the household. Further, more than one third of respondents (35.4%) indicated at least one chronic health condition.

Bivariate analysis (Table 2) showed that all health, risk, and individual characteristics were independently associated with the outcome food access except for having a chronic health condition. Respondents with job impacts, poor health outcomes, high-risk households, and essential workers reported worse food access. Respondents that were Hispanic, female (as well as non-binary or other gender), younger, and less educated also had worse food access. Males and non-Hispanic White respondents fared better with food access. All variables independently associated with the outcome food access were examined in a multivariate analysis (Table 3).

In model one, the unadjusted association between reduced work due to the pandemic was examined with the outcome food access. Respondents that reported reduced work were 1.8 times more likely to report worse food access without accounting for any additional experiences or characteristics. In model two, COVID-19 health impact variables were added. Reduced work due to the pandemic remained a significant predictor of variance in the outcome, with respondents reporting reduced work due to the pandemic having 1.6 times greater odds of worse food access (OR 1.58, 95% CI 1.03, 2.42) than respondents without a negative job impact. Health factors were also significant, with participants that screened positive for likely Major Depressive Disorder having more than twice the odds of worse food access (aOR 2.27, 95% CI 1.32, 3.91) and respondents reporting a direct COVID-19 impact having more than 1.5 times the odds for worse food access (aOR 1.61, 95% CI 1.03, 2.52).

Model three added risk factors for COVID-19 including race/ethnicity, living in a high-risk household, and being an essential worker. In model three, reduced work and direct COVID-19 impact were explained away. Likely Major Depressive Disorder persisted in explaining variance in the outcome food access (aOR 3.49, 95% CI 2.23, 5.46), and Hispanic ethnicity and the essential worker risk group arose as explanatory of the difference in reported food access. Study participants that screened positive for Major Depressive Disorder had 3.5 times greater odds of worse food access than those reporting better mental health. Hispanic respondents had more than twice the odds of worse food access compared to non-Hispanic White participants (aOR 2.52, 95% CI 1.37, 4.62), and essential workers had nearly twice the odds of worse food access compared to non-essential workers (aOR 1.69, 95% CI 1.07, 2.67).

In the final model, individual characteristics were added. Likely Major Depressive Disorder, Hispanic ethnicity, and essential worker status continued to explain variance in the outcome food access after accounting for individual characteristics regardless of any individual characteristics including gender, income prior to the pandemic, or age.

## 4. Discussion

Few studies have examined the impact of COVID-19 on food access, and no study has examined the relationship between high-risk groups, mental health, and food access. This study examined the direct (i.e., COVID-19 illness and mental health) and indirect (i.e., work disruption, high-risk household, and essential worker status) impacts of COVID-19 on food access in New York State. Findings showed that Hispanic ethnicity, essential worker status, and likely Major Depressive Disorder were associated with reduced food access after adjusting for individual characteristics, employment and health impacts, and high risk for exposure or severe illness. Although bivariate analysis showed differences in food access by individual characteristics such as age, gender, education, and income prior to the pandemic, these characteristics did not vary based on food access status in multivariate models, which suggests that food access is a problem regardless of demographic (except for Hispanic ethnicity).

The COVID-19 pandemic exacerbated food access problems for low-income households and communities of color who already experience higher rates of food insecurity and chronic conditions that put them at higher risk of severe illness [27,43,44]; limited research on COVID-19 impacts on food access and food insecurity show that communities of color, and particularly non-Hispanic Black and Hispanic Americans, have been most affected [44,45]. These findings are similar to research prior to the pandemic, which showed that BIPOC experience higher rates of food access issues and food insecurity [7,46]. BIPOC are also more likely to live in neighborhoods with lower access to food and to work essential jobs, which have been expected to put individuals at higher risk for infection and food access issues, in part due to their low pay, inflexibility, and lack of paid sick leave [43,44,47]. This research supports these conclusions and furthers the research by showing that essential workers are experiencing worse food access during COVID-19.

COVID-19 has been projected to increase mental health disorders such as anxiety and depression among those who have been infected with the virus or know somebody infected with the virus as well as among those experiencing job loss, disruption, or uncertainty [48,49]. Higher rates of mental health disorders, including depression, have also been reported among food-insecure individuals [50,51]. This suggests that respondents reporting worse food access in this study may be more likely to suffer from depression due, in part, to their limited food access. 

The findings in this study differ from those of other research that has reported relationships between food access or food insecurity and demographic/socioeconomic characteristics including income, age, education, job loss and disruption, and gender during the pandemic [27,44]. These studies did not include essential workers or mental health in their analyses. Accounting for these factors may help to explain issues with food access experienced at this time; essential workers may better capture the financial burden on low-wage workers and their likelihood of experiencing work disruptions. Additionally, our sample included low-income and BIPOC New York State residents whose experience may differ from that of residents of other states and that of the population as a whole.

This study has several limitations to bear in mind when interpreting the results. Data were collected using a cross-sectional study design; therefore, analyses are not able to determine casual relationships. To address this limitation, respondents were asked to provide their perception of the impact of the pandemic or information about before and since the pandemic to characterize COVID-19 effects. The outcome of interest, food access, was collected cross-sectionally and therefore examined only since the beginning of the pandemic, so it is unclear if respondents were experiencing food access issues prior to the pandemic. Respondents experiencing worse food access may have been experiencing it before the pandemic began, which limits this study’s ability to conclude that COVID-19 led to reductions in food access. The study oversampled New York State residents with greater risk related to COVID-19. As a result of oversampling, generalizations cannot be made about all New Yorkers, including New York City residents. However, examining the experiences of individuals with greater risk of COVID-19 and complications if infected is important given the documented disparities during the pandemic. Finally, survey data were collected through a web platform, and therefore, access to the internet was a requirement for all study participants. This method was selected to facilitate timely data collection during the phased reopening from NY on Pause. Web data collection systematically excludes anyone without access to the internet. Although not ideal, this approach provides important information about the experiences of New York State residents during ongoing measures to reduce the spread of COVID-19, and research shows that most Americans have internet access across racial, ethnic, and income groups [52]. 

Additional research with alternate methods is needed to understand additional experiences throughout the course of the pandemic, populations with limited internet access, and how food access during the pandemic influences long-term health. Given the populations reporting worse food access in this study, policy solutions may include mandating hazard pay, paid sick leave, and childcare support for essential workers. Further, increased participation of BIPOC in public health policy is crucial. Pandemic planning requirements at the state and federal level could require input from diverse stakeholders and fund programs that aim to reduce disparities in food access. Ongoing pandemic relief legislation may also consider additional financial supports for these groups.

## 5. Conclusions

Hispanic residents of New York State, those working essential jobs, and those with likely Major Depressive Disorder were more likely to experience issues with food access during COVID-19. BIPOC make up a higher percentage of essential workers, and Hispanic individuals and essential workers in particular have been affected by mental health issues at disproportionately high rates during COVID-19 [53]. Essential workers have faced increased risk of exposure to the virus and to limited food access due to their work in low-wage, inflexible jobs that often do not offer paid sick leave. While no different from the risk associated with food access prior to the pandemic, these individuals are likely in greater need of support during this time, particularly because of the pandemic’s economic consequences. Additional support for this group to meet their food needs is important for a sustainable response to a prolonged pandemic event. Nutrition is important for health and wellbeing, and failing to provide adequate food assistance to essential workers will create downstream problems with illness, absenteeism, and burnout. Providing adequate assistance will ensure that the U.S. is working toward meeting several United Nations Sustainable Development goals, including achieving food security and improved nutrition (goal 2), promoting health and wellbeing (goal 3), reducing inequality (goal 10), and building inclusive and accountable institutions (goal 16) [54].

## Figures and Tables

**Table 1:** Sample characteristics.

Characteristics	Frequency	Percent
**Gender**		
Female, non-binary, other	235	56.6
Male	180	43.4
**Income in 2019**		
<$13,000	74	17.8
$13,000–24,999	74	17.8
$25,000–49,999	106	25.5
$50,000–74,999	63	15.2
$75,000+	98	23.6
**Age**		
18–24	128	30.8
25–44	155	37.4
45–64	88	21.2
65+	44	10.6
**Education**		
High school or less	131	31.6
Some college, Associate’s degree, technical school	158	38.1
Bachelor’s or graduate degree	126	30.4
**Race/Ethnicity**		
Non-Hispanic White	82	20.6
Black or African American	126	31.6
Hispanic	162	40.6
Other, more than one race	29	7.3
Reduced work	169	40.7
Likely Generalized Anxiety Disorder	164	39.5
Likely Major Depressive Disorder	171	41.2
Direct COVID-19 Impact	258	62.2
Self COVID-19 Impact	84	20.2
Essential worker	151	36.4
High-Risk Household (65+, chronic health condition)	171	41.2
Chronic condition	147	35.4

**Table 2:** Sample characteristics and factors independently associated with food access (chi2).

Factors	Better Food Access		Worse Food Access		*p*-Value
	Frequency	Percent	Frequency	Percent	
Reduced work	59	32.6	110	47	*p* < 0.01
Likely Generalized Anxiety Disorder	45	24.9	119	50.9	*p* < 0.000
Likely Major Depressive Disorder	46	25.4	125	53.4	*p* < 0.000
Direct COVID-19 Impact	97	53.6	161	68.8	*p* < 0.01
Self COVID-19 Impact	28	15.5	56	23.9	*p* < 0.05
**Race/Ethnicity**					
Non-Hispanic White	51	28.5	31	14.1	*p* < 0.01
Black or African American	58	32.4	68	30.9	
Hispanic	58	32.4	104	47.3	
Other, more than one race	12	6.7	17	7.7	
Essential worker	55	30.4	96	41	*p* < 0.05
High-Risk Household (65+, chronic health condition)	63	34.8	108	46.2	*p* < 0.05
Chronic condition	57	31.5	90	38.5	*p* = 0.141
**Gender**					
Female, non-binary, other	88	48.6	147	62.8	*p* < 0.01
Male	93	51.4	87	37.2	
**Income in 2019**					
<$13,000	34	18.8	40	17.1	*p* < 0.05
$13,000–24,999	22	12.2	52	22.2	
$25,000–49,999	42	23.2	64	27.4	
$50,000–74,999	31	17.1	32	13.7	
$75,000+	52	28.7	46	19.7	
**Age**					
18–24	50	27.6	78	33.3	*p* < 0.000
25–44	54	29.8	101	43.2	
45–64	39	21.6	49	20.9	
65+	38	21	6	2.6	
**Education**					
High school or less	49	27.1	82	35	*p* < 0.01
Some college, Associate’s degree, technical school	62	34.3	96	41	
Bachelor’s or graduate degree	70	38.7	56	23.9	

**Table 3:** Factors associated with worse food access (logistic regression).

Factors	Model 1: Employment Impact		Model 2: Health Impact		Model 3: COVID-19 Risk		Model 4: Individual Characteristics	
	OR	95% CI	aOR	95% CI	aOR	95% CI	aOR	95% CI
Reduced work	**1.83**	**1.23, 2.75**	**1.58**	**1.03, 2.42**	1.46	0.94, 2.28	1.15	0.72, 1.84
Likely Generalized Anxiety Disorder			1.7	0.98, 2.94	-	-	-	-
Likely Major Depressive Disorder			**2.27**	**1.32, 3.91**	**3.49**	**2.23, 5.46**	**2.95**	**1.83, 4.77**
Direct COVID-19 Impact			**1.61**	**1.03, 2.52**	1.43	0.92, 2.23	1.44	0.89, 2.32
Self COVID-19 Impact			1.26	0.72, 2.22	-	-	-	-
**Race/Ethnicity**								
Non-Hispanic White					ref			
Black or African American					1.62	0.87, 3.01	1.21	0.60, 2.45
Hispanic					**2.52**	**1.37, 4.62**	**1.87**	**1.05, 3.86**
Other, more than one race					2.17	0.86, 5.47	1.53	0.58, 4.10
Essential worker					**1.69**	**1.07, 2.67**	**1.66**	**1.04, 2.65**
High-Risk Household (65+, chronic health condition)					1.52	0.97, 2.37	1.57	0.99, 2.50
**Gender**								
Female, non-binary, other							ref	
Male							0.87	0.54, 1.40
**Income in 2019**								
<$13,000							ref	
$13,000–24,999							2.19	1.00, 4.82
$25,000–49,999							1.57	0.77, 3.20
$50,000–74,999							1.18	0.52, 2.64
$75,000+							1.27	0.57, 2.79
**Age**								
18–24							ref	
25–44							1.69	0.96, 2.98
45–64							1.35	0.71, 2.57
65+							0.27	0.09, 0.78
**Education**								
High school or less							ref	
Some college, Associate’s degree, technical school							0.79	0.45, 1.39
Bachelor’s or graduate degree							0.54	0.28, 1.02
Log Likelihood	−279.8		−258.7		−241.7		−227.5	
AIC	563.7		529.4		501.3		493.0	
BIC	571.7		553.6		537.2		568.8	

OR: Odds ratio; aOR: Adjusted Odds Ratio; 95% CI: 95% Confidence Interval; ref: reference group; AIC: Akaike Information Criterion; BIC: Bayesian Information Criterion; bold text: statistically significant at *p* < 0.05 level.

## Data Availability

The data presented in this study are available on request from the corresponding author.
